# Increased myocardial native T1 and extracellular volume in patients with Duchenne muscular dystrophy

**DOI:** 10.1186/s12968-016-0224-7

**Published:** 2016-01-21

**Authors:** Jonathan H. Soslow, Stephen M. Damon, Kimberly Crum, Mark A. Lawson, James C. Slaughter, Meng Xu, Andrew E. Arai, Douglas B. Sawyer, David A. Parra, Bruce M. Damon, Larry W. Markham

**Affiliations:** 1 Thomas P Graham Jr. Division of Pediatric Cardiology, Department of Pediatrics, Monroe Carell Jr. Children’s Hospital, Vanderbilt University Medical Center, 2200 Children’s Way Suite 5230, Doctors’ Office Tower, Nashville, TN 37232-9119 USA; 2 Department of Electrical Engineering and Computer Sciences, Vanderbilt University, Nashville, TN USA; 3 Division of Cardiovascular Medicine, Department of Medicine, Vanderbilt University Medical Center, Nashville, TN USA; 4 Department of Biostatistics, Vanderbilt University Medical Center, Nashville, TN USA; 5 National Heart, Lung and Blood Institute (NHLBI), National Institutes of Health (NIH), Bethesda, MD USA; 6 Department of Cardiac Services, Maine Medical Center, Portland, ME USA; 7 Department of Radiology and Radiological Sciences, Vanderbilt University Medical Center, Nashville, TN USA; 8 Department of Molecular Physiology and Biophysics, Vanderbilt University, Nashville, TN USA; 9 Department of Biomedical Engineering, Vanderbilt University, Nashville, TN USA

**Keywords:** Duchenne muscular dystrophy, Cardiomyopathy, Cardiovascular magnetic resonance, Extracellular volume fraction, T1 mapping

## Abstract

**Background:**

Duchenne muscular dystrophy (DMD) cardiomyopathy is a progressive disease for which there is no cure. Disease-specific therapies are needed that can be initiated before irreversible myocardial damage ensues. In order to evaluate therapeutic efficacy, surrogate endpoints other than ejection fraction must be found. The hypothesis of this study is that T1 and extracellular volume fraction (ECV) mapping using cardiovascular magnetic resonance (CMR) can detect diffuse extracellular matrix expansion in DMD patients with normal left ventricular ejection fraction (LVEF) and without myocardial late gadolinium enhancement (LGE).

**Methods:**

Thirty-one DMD and 11 healthy control participants were prospectively enrolled. CMR using a modified Look-Locker (MOLLI) sequence was performed in all participants before and after contrast administration. T1 and ECV maps of the mid left ventricular myocardium were generated and regions of interest were contoured using the standard 6-segment AHA model. Global and segmental values were compared between DMD and controls using a Wilcoxon rank-sum test.

**Results:**

The DMD participants had significantly higher mean native T1 compared with controls (1045 ms vs 988 ms, *p* = 0.001). DMD participants with normal LVEF and without evidence of LGE also demonstrated elevated mean native T1 (1039 ms vs 988 ms, *p* = 0.002, and 1038 ms vs 988 ms, *p* = 0.011). DMD participants had a significantly greater mean ECV than controls (0.31 vs 0.24, *p* < 0.001), even in the settings of normal LVEF (0.28 vs 0.24, *p* < 0.001) and negative LGE (0.29 vs 0.24, *p* = 0.001).

**Conclusions:**

DMD participants have elevated LV myocardial native T1 and ECV, even in the setting of normal LVEF and in the absence of LGE. T1 and ECV mapping in DMD have potential to serve as surrogate cardiomyopathy outcome measures for clinical trials.

**Electronic supplementary material:**

The online version of this article (doi:10.1186/s12968-016-0224-7) contains supplementary material, which is available to authorized users.

## Background

Duchenne muscular dystrophy (DMD) affects 1 in 4700 live male births [1]. DMD is a progressive disease leading to skeletal and cardiac myopathy. The development of cardiomyopathy, defined here as abnormal ejection fraction, occurs in the majority of boys with DMD by 18 years of age [2]. With improvements in supportive care measures that mitigate the complications of skeletal muscle disease, cardiovascular disease has become the leading cause of death [3]. Standard heart failure medications, such as angiotensin converting enzyme inhibitors and beta-blockers, confer some benefits, but ultimately only delay the inexorable decline in cardiac function [4, 5]. More effective DMD-specific therapies are necessary to reduce the morbidity and mortality associated with DMD cardiomyopathy [5]. Evaluation of therapeutic efficacy, however, is difficult. Assuming a study treatment is initiated early enough in the disease course to have maximal effect, it could take over a decade of therapy to reach traditional study trial endpoints such as reduced left ventricular ejection fraction (LVEF). Earlier, non-invasive biomarkers of cardiac disease progression are integral to the ongoing clinical care of these patients and to the evaluation of therapeutic alternatives for DMD.

Pathological studies demonstrate significant fibrosis in DMD myocardium that usually begins in the subepicardium of the left ventricular (LV) free wall [6]. Late gadolinium enhancement (LGE) using cardiovascular magnetic resonance (CMR) can reveal extracellular matrix (ECM) expansion consistent with fibrosis in the same location, [7, 8] but quantification of LGE in DMD can be difficult [9]. LGE is best for detection of focal areas of ECM expansion, not the diffuse ECM expansion found in boys with DMD [10–12]. In addition, by the time larger areas of ECM expansion are detectable by LGE, it may be too late to reverse the myocardial damage.

CMR mapping of the myocardial longitudinal relaxation time constant, T1, can reveal myocardial abnormalities such as fibrosis and edema. Previously, we used a retrospective analysis to report elevated post-contrast T1 times in boys with DMD [13]. T1 measurements can be used to calculate the myocardial extracellular volume fraction (ECV), a quantitative technique that allows estimation of ECM expansion [14]. Pathological studies have demonstrated a strong correlation between ECV and ECM expansion caused by fibrosis and edema [12, 15–17]. Studies have reported elevated ECV in multiple myocardial disease processes and ECV can predict mortality [18, 19]. A single study in a cohort of Becker muscular dystrophy patients demonstrated elevated ECV when compared with controls [20]. However, to our knowledge, no published studies have evaluated ECV in patients with DMD. We hypothesized that the diffuse ECM expansion found in DMD is detectable using T1 and ECV mapping, even in the absence of standard measures of myocardial disease such as abnormal LVEF and LGE.

## Methods

### Patient selection

This single-center, prospective, observational study was approved by the Vanderbilt University Institutional Review Board and completed between January 2013 and July 2015. Consent was obtained for all participants; those under 18 years of age signed an age-appropriate assent form. Participants in the DMD group were recruited from the multidisciplinary Neuromuscular-Cardiology Clinic and were over 7 years of age. The diagnosis of DMD was confirmed by either skeletal muscle biopsy or the presence of a dystrophin mutation and skeletal muscle weakness. Exclusion criteria were: requiring sedation for CMR, renal dysfunction or other contraindication to contrast-enhanced CMR.

Healthy participants aged 18–30 with a body mass index ranging from 18 to 25 kg/m^2^ were recruited into the control group. Exclusion criteria were: cardiovascular disease (including but not limited to congenital heart disease, cardiomyopathy, secondary heart disease such as rheumatic fever or Kawasaki’s disease), risk factors for cardiovascular disease (such as arrhythmia or hypertension), muscular dystrophy or unexplained skeletal muscle weakness, any diagnosis that could affect cardiac function or lead to myocardial fibrosis, and renal dysfunction (glomerular filtration rate <75 ml/min assessed on day of scan) or other contraindication to CMR with gadolinium.

Two DMD participants were excluded for inadequate images. Two control participants were excluded for abnormal CMRs (bicuspid aortic valve and pericardial effusion) and one was excluded for inadequate images.

### Study procedure

Pertinent DMD clinical data were collected from the electronic medical record. For the control group, a phone screening was performed to assess suitability, including comprehensive questioning on past and family medical history. Prior to the CMR, the participant completed a health risk assessment. All participants provided a blood sample for measurement of hematocrit on day of CMR.

### Cardiovascular magnetic resonance

CMR was performed on a 1.5 Tesla Siemens Avanto (Siemens Healthcare Sector, Erlangen, Germany) with an 8 channel cardiac coil. Functional imaging was performed as previously described using balanced steady-state free precession (bSSFP) imaging [13]. Breath-held, T2-weighted (effective TE 60 ms) double inversion turbo spin-echo imaging was performed prior to contrast administration.

The control group and a subset of DMD participants underwent T2 mapping using a breath-held, electrocardiogram (ECG)-triggered, bSSFP sequence with motion correction. Typical imaging parameters were as follows: Adiabatic T2 preparation with 35° flip angle, field of view 340 × 272 mm^2^, matrix size 192 × 144, slice thickness 8 mm, voxel size 1.8 × 1.9 × 8.0 mm^3^, TR/TE 2.5 ms/1.1 ms, parallel imaging factor of 2. Intravenous Gd-DTPA contrast (gadopentate dimeglumine, Magnevist®, Bayer Healthcare Pharmaceuticals, Wayne, NJ, USA) was administered through a peripheral intravenous line (PIV) at a dose of 0.2 mmol/kg. Multiple LGE techniques were used to insure detection of enhancement: 1) single shot and 2) segmented inversion recovery bSSFP with optimized inversion recovery to null the signal from the myocardium, and 3) phase sensitive inversion recovery bSSFP with an inversion time of 300 ms.

Breath-held modified Look-Locker inversion recovery (MOLLI) sequences were performed prior to and 15 min after contrast administration in the mid-ventricular level in the short axis plane at the same slice location as the T2 mapping and the T2-weighted, cine, and LGE imaging [10, 21]. MOLLI sequences were motion-corrected, ECG-triggered images obtained in diastole with typical imaging parameters: non-selective inversion with a 35° flip angle, single shot SSFP imaging, initial inversion time of 120 ms with 80 ms increments, field of view 340 × 272 mm^2^, matrix size 256 × 144, slice thickness 8 mm, voxel size 1.3 × 1.9 × 8.0 mm^3^, TR/TE 2.6 ms/1.1 ms, parallel imaging factor of 2. The matrix size was decreased to 192 × 128 for heart rates >90 (approximate voxel size 1.8 × 2.1 × 8 mm^3^). The initial pre-contrast MOLLI acquired 5 images after the first inversion with a 3 beat pause followed by 3 images after the second inversion, or 5(3)3. This was later modified to a 3 s pause, or 5(3 s)3, to reduce bias from higher heart rates; the post-contrast protocol was acquired at a 4(1)3(1)2 [22]. Motion correction as described by Xue, et al. was performed and a T1 map was generated on the scanner [23]. A goodness of fit map was also performed in most participants at the time of the scan to evaluate data quality. One investigator (JS) was present for all scans and reviewed the MOLLI sequences with the technologist for adequacy at the time of the scan. Any image felt to be inadequate due to poor breath holds or poor motion correction was repeated at the time of the scan.

### Image processing

One reader (JS) traced all contours in this study. LV volume, mass, and function were calculated as previously described [13]. The presence or absence of LGE was qualitatively assessed by one reader and confirmed by a second reader. T2 maps were contoured to obtain global myocardial T2.

**Fig. 1:**
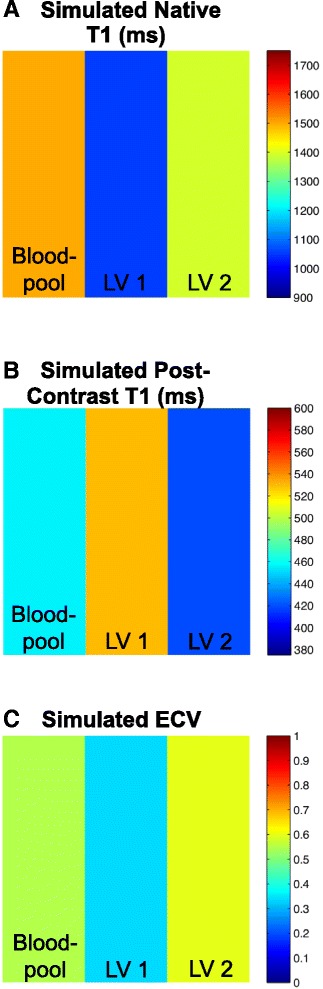
Validation of T1 and ECV mapping software. To confirm that our results were quantitatively correct, we generated a simulated dataset for both pre and post-contrast data. In this dataset, we use inversion times of 120, 1018, 1698, 3125 milliseconds and 280, 693, 755, 1860 milliseconds for pre and post-contrast data respectively. A hematocrit value of 0.45 was used. Three different pseudo regions of interest (ROIs) were generated to simulate the blood pool and two left ventricular ROIs. This simulated data is generated such that we expect the following T1 times for the following regions: T1pre_bloodpool = 1498.3 ms, T1pre_LV1 = 1054.7 ms and T1pre_LV2 = 1393.6 ms, T1post_bloodpool = 419.7 ms, T1post_LV1 = 529.7 ms, and T1post_LV2 = 419.7 ms. We also expect ECV values for the following regions to be 0.55, 0.33, and 0.60 respectively. The results in (**a**), (**b**), and (**c**) match with the a priori defined dataset

T1 maps, obtained prior to and after contrast administration as described by Messroghli et al., [21] were used along with the subject’s hematocrit to calculate an ECV map using software programmed in MATLAB 2014a (The MathWorks, Natick, MA, USA). Affine two-dimensional image registration was performed using a normalized mutual information cost and search function. Native and post-contrast T1 maps were registered to the native T1 map. The field of view was cropped to the region of interest to prevent spurious maximization of mutual information that would result in poor alignment. In cases of suboptimal T1 map image registration on the scanner, the sequence was either repeated without motion correction or image registration was performed using MATLAB software. T1 maps were computed from magnitude reconstructed MR data using iterative sign inversion Levenberg-Marquardt optimization, as complex data were not available. This process results in multiple optimizations and T1 estimates per voxel. The best fit, and therefore best estimate of T1, was determined by the optimization that had the smallest root mean squared error. Affine two-dimensional image registration was again used and all image frames, both pre and post-contrast, were registered to the first pre-contrast frame for consistency. The T1 and ECV calculations were validated using an a priori defined dataset (Fig. 1). The ECV was calculated as:

$$ \mathrm{E}\mathrm{C}\mathrm{V}=\frac{\left(\frac{1}{\mathrm{myocardialT}1\mathrm{p}\mathrm{ost}}\right)-\left(\frac{1}{\mathrm{myocardialT}1\mathrm{p}\mathrm{r}\mathrm{e}}\right)}{\left(\frac{1}{\mathrm{bloodpoolT}1\mathrm{p}\mathrm{ost}}\right)-\left(\frac{1}{\mathrm{bloodpoolT}1\mathrm{p}\mathrm{r}\mathrm{e}}\right)}\left(1-\mathrm{H}\mathrm{c}\mathrm{t}\right) $$

**Fig. 2:**
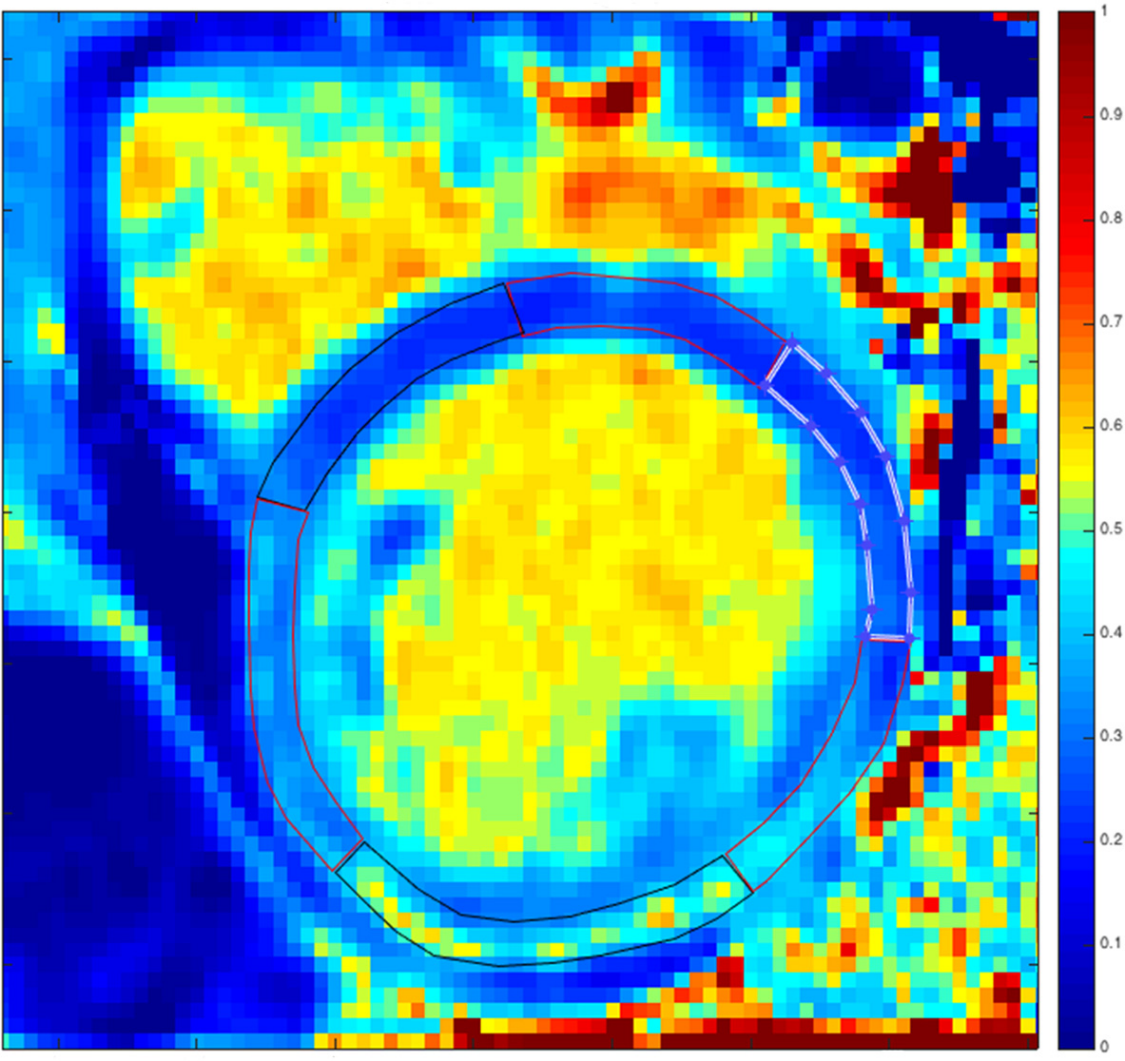
Example regions of interest. Example regions of interest drawn on extracellular volume map in a Duchenne muscular dystrophy participant with late gadolinium enhancement primarily in the subepicardium of the free wall

Regions of interest (ROIs) were manually drawn on T1 and ECV maps within the LV mesocardium in 6 segments using the standard AHA/ACC model of segmentation (Fig. 2) [24]. Global myocardial T1 and ECV values were calculated from segmental values. ROIs were carefully traced to avoid partial volume averaging with blood-pool or epicardial fat. Based on the T1 mapping consensus statement, areas of LGE were included in the ROIs as these areas were felt to be the most focal areas in a continuum of diffuse ECM expansion [10]. For cases where the ROIs were difficult to trace, LGE images were used to help trace the ROIs. Imaging artifact was not contoured. Segments were not included in the analysis if the bounds of the myocardium could not be distinguished from surrounding tissue and blood pool or if image registration was inadequate in those segments. In order to assess the ECV of large areas of LGE, targeted ROIs were traced in areas of clear LGE on ECV maps. These ROIs were confirmed by a second reader (DP).

A random sample of 10 ECV maps was re-contoured by a separate reader (DP) to assess inter-observer variability. The same sample was re-contoured by the initial reader one month later to assess intra-observer variability.

The quality of each ECV map was graded using methods reported by Kellman et al. [14] whereby ECV maps were assigned a score ranging from 1 to 5, with 1 representing a non-diagnostic map and 5 representing an excellent map. None of the maps with a score less than 3 were included.

### Statistical analysis

Demographic variables were compared using either a Wilcoxon rank-sum (continuous variables) or a Chi-square or Fisher’s exact test (categorical variables). Inter- and intra-observer variability were assessed using an intraclass correlation coefficient. Correlations between continuous variables were evaluated using Spearman’s rho (2 variables) or linear regression (controlling for multiple variables). The segmental and global native T1 and ECV were compared between groups using Wilcoxon rank-sum tests. Subset analyses included comparisons of DMD participants with normal LVEF (≥55 %) and without LGE to controls. The variance of ECV measurements within each segment was calculated by squaring the standard deviation. We then averaged the variances from each segment and took the square root, which estimates the average within-subject standard deviation.

Analyses were performed with IBM SPSS statistics, version 22.0 (Armonk, NY: IBM Corp). Study data were collected and managed using REDCap (Research Electronic Data Capture) electronic data capture tools hosted at Vanderbilt [25].

## Results

### Demographics

The study enrolled 31 DMD and 11 healthy participants. The DMD participants were younger than the controls (Table 1). DMD participants had faster heart rates and were shorter and weighed less, resulting in smaller body surface areas. There were no other significant demographic differences.

**Table 1:** Demographics

	Control	DMD^a^
	*N* = 11	*N* = 31
Age (years)	24.5 ± 3.9 (range 18–30)	13.4 ± 4.7 (range 8–27)
Male Gender (%)	100 %	100 %
Height (cm)	182 ± 8	144 ± 15
Weight (kg)	76 ± 9	51 ± 16
BSA^b^ (m^2^)	2.0 ± 0.2	1.4 ± 0.3
BMI^c^ (kg/m^2^)	23.0 ± 1.5	24.1 ± 6.3
Heart Rate (bpm)	66 ± 4	94 ± 14
Ambulatory (%)	11 (100 %)	7 (22.6 %)
Ventilatory support (%)	0 %	7 (22.6 %)
Race		
Caucasian	8 (72.7 %)	27 (87.1 %)
African American	1 (9.1 %)	2 (6.5 %)
Asian	1 (9.1 %)	2 (6.5 %)
Other	0	0
Mixed	1 (9.1 %)	0
Hispanic/Latino	1 (9.1 %)	3 (9.7 %)
Medical Therapy at CMR^d^		
ACEi^e^	0 %	14 (45.2 %)
ARB^f^	0 %	5 (16.1 %)
β-blocker	0 %	10 (32.3 %)
Corticosteroids	0 %	21 (67.7 %)
Corticosteroid duration (years)	0	3.7 ± 3.2

^a^Duchenne muscular dystrophy (DMD)

^b^Body surface area (BSA)

^c^Body mass index (BMI)

^d^Cardiovascular Magnetic Resonance (CMR)

^e^Angiotensin Converting Enzyme Inhibitor (ACEi)

^f^Angiotensin Receptor Blocker (ARB)

### LV volume and function

The DMD group had a lower LVEF than controls (54.8 % ± 8.1 vs 60.3 % ± 2.7, *p* = 0.033) (Table 2). All control participants had normal LVEF (≥55 %). Twelve of the 31 DMD participants (38.7 %) had abnormal LVEF. All DMD participants with abnormal LVEF had regional wall motion abnormalities; 21 DMD participants had regional wall motion abnormalities. The DMD group had a smaller indexed left ventricular end diastolic volume (63.8 ml/m^2^ ± 14.8 vs 79.3 ml/m^2^ ± 11.0, *p* = 0.001). This is consistent with the minimal early LV dilatation previously reported in DMD [26]. There were no other significant differences in chamber dimensions.

**Table 2:** Cardiac MRI findings in control and Duchenne muscular dystrophy (DMD) participants

	Control	DMD^a^	*p*-value
	*N* = 11	*N* = 31	
LVEF^b^ (%)	60.3 ± 2.7	54.8 ± 8.1	0.033
Indexed LVEDV^c^ (ml/m^2^)	79.3 ± 11.0	63.8 ± 14.8	0.001
Indexed LVESV^d^ (ml/m^2^)	33.2 ± 4.8	29.4 ± 11.1	0.062
Indexed LV mass (g/m^2^)	58.3 ± 8.0	53.2 ± 10.1	0.058
RVEF^e^ (%)	56.1 ± 3.4	56.6 ± 5.1	0.552
LGE^f^ abnormal	0	19 (61.3 %)	<0.001^#^
Basal Anterior	0	4 (12.9 %)	
Basal Anteroseptal	0	1 (3.2 %)	
Basal Inferoseptal	0	3 (9.7 %)	
Basal Inferior	0	12 (38.7 %)	
Basal Inferolateral	0	18 (58.1 %)	
Basal Anterolateral	0	18 (58.1 %)	
Mid Anterior	0	6 (19.4 %)	
Mid Anteroseptal	0	3 (9.7 %)	
Mid Inferoseptal	0	5 (16.1 %)	
Mid Inferior	0	13 (41.9 %)	
Mid Inferolateral	0	17 (54.8 %)	
Mid Anterolateral	0	16 (51.6 %)	
Apical Anterior	0	5 (16.1 %)	
Apical Septal	0	6 (19.4 %)	
Apical Inferior	0	5 (16.1 %)	
Apical Lateral	0	7 (22.6 %)	
Apex	0	3 (9.7 %)	

^#^Fisher’s exact test (rest Wilcoxon rank-sum)

^a^Duchenne muscular dystrophy (DMD)

^b^Left ventricular ejection fraction (LVEF)

^c^Left ventricular end diastolic volume (LVEDV)

^d^Left ventricular end systolic volume (LVESV)

^e^Right ventricular ejection fraction (RVEF)

^f^Late gadolinium enhancement (LGE)

### Late gadolinium enhancement

No control patients had LGE. Nineteen of the 31 DMD participants had LGE in at least one segment (Table 2); 16 of those participants had LGE in the mid-ventricular slice used for T1 mapping. Eighteen of the 19 DMD participants with LGE had regional wall motion abnormalities. DMD participants with LGE had significantly lower LVEF than DMD without LGE (51.5 % ± 8.6 vs 60.2 % ± 2.7, *p* = 0.001). All 12 DMD participants with abnormal LVEF had at least one segment with LGE. LGE was present in 7 DMD participants with normal LVEF.

### T2-weighted images and T2 mapping

No significant edema was detected by subjective analysis of T2-weighted imaging. All controls and 12 DMD participants underwent T2 mapping. The mean T2 values for both DMD and control participants were not suggestive of myocardial edema and were lower in DMD compared with control (47 ms ± 2 vs 49 ms ± 1, *p* < 0.001).

### T1 mapping and extracellular volume

For native T1 maps, 99.2 % of segments were contoured; 99.6 % of segments on ECV maps were contoured. For the ROIs of LGE performed on ECV maps, 15 of 19 had ROIs contoured. The remaining 4 either had no LGE at that particular slice location or had such a small area of LGE that contouring was not possible. The quality of DMD ECV maps was excellent in 15 participants (48.4 %), good in 13 participants (41.9 %), and adequate in 3 participants (9.7 %). The ECV quality in controls was excellent in 7 participants (63.6 %) and good in 4 participants (36.4 %). The ECV map quality did not differ significantly between DMD and controls (*p* = 0.475).

**Fig. 3:**
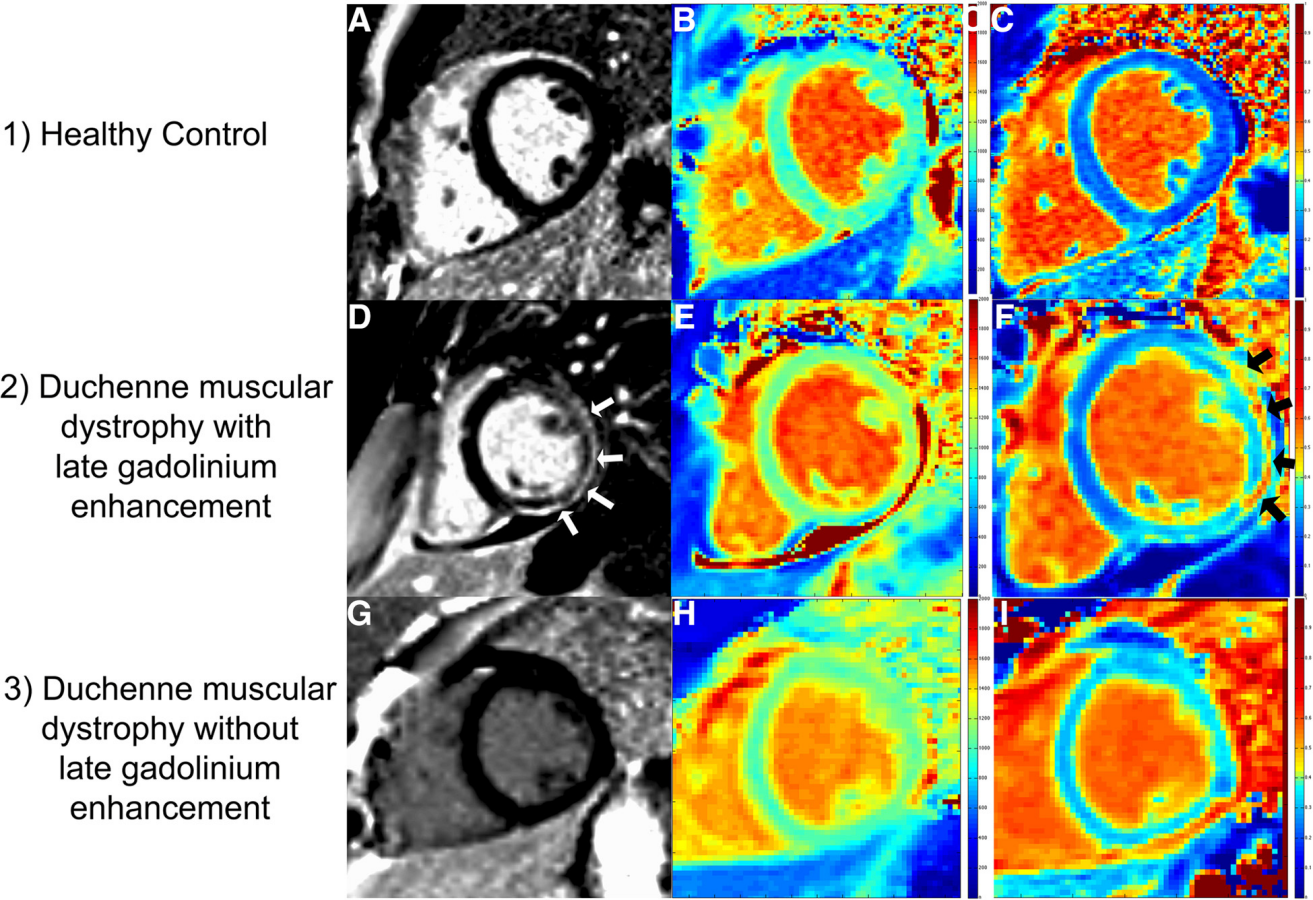
Example cardiac MRI images. 1) Healthy control without late gadolinium enhancement (LGE) (**a**), normal native T1 (**b**), and ECV (**c**) values. 2) Duchenne muscular dystrophy (DMD) participant with significant LGE (**d**) (arrows); native T1 map (**e**) demonstrates increased T1; ECV map (**f**) demonstrates similar areas of elevated ECV (arrows) as the LGE. 3) LGE image in DMD participant without LGE (**g**) but with diffusely elevated native T1 (**h**) and ECV (**i**) suggesting extracellular matrix expansion

**Fig. 4:**
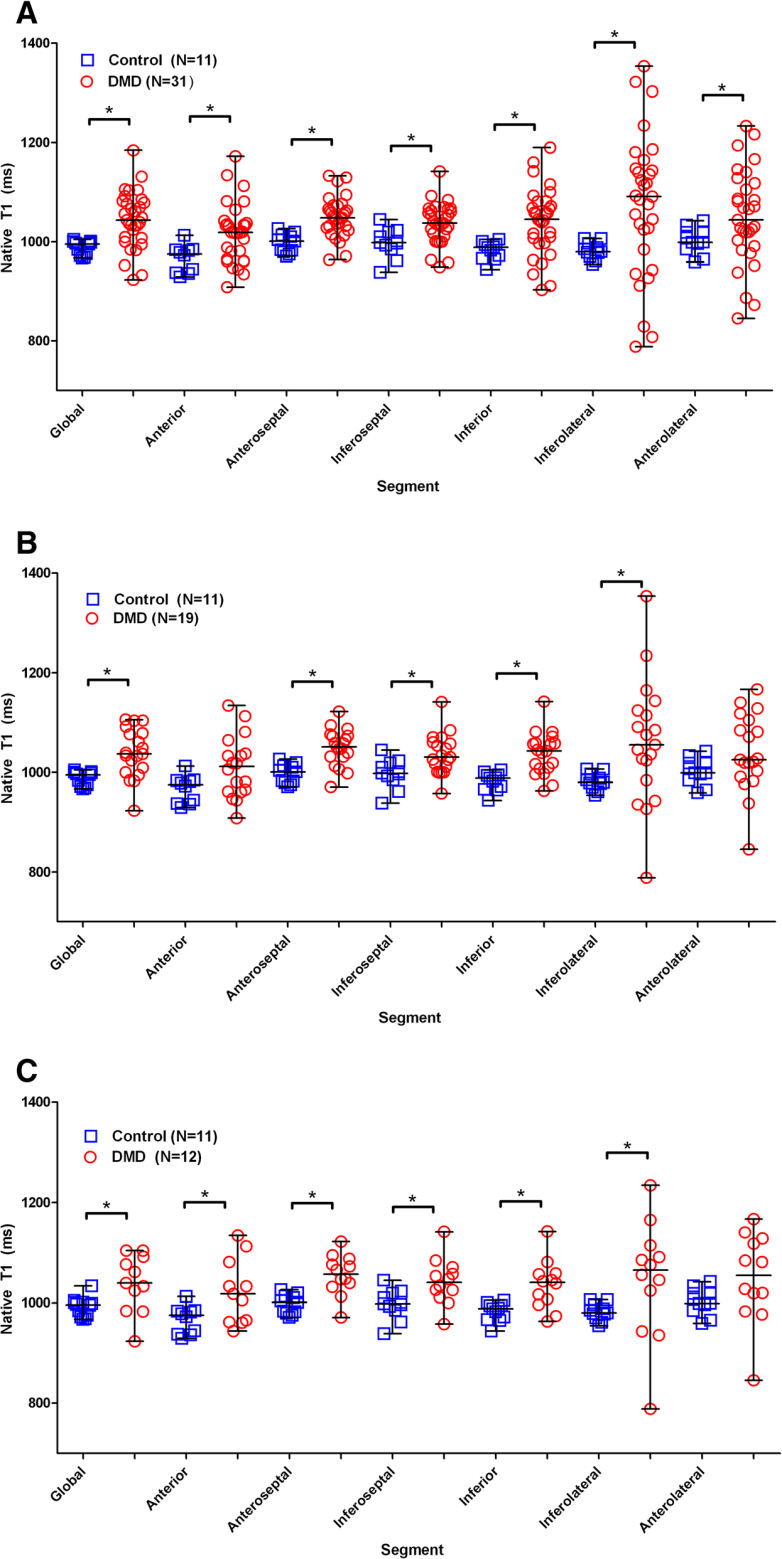
Scatterplots of global and segmental native T1 values in Duchenne Muscular Dystrophy (DMD) and controls. (**a**) All DMD participants compared with controls, (**b**) DMD participants with normal left ventricular ejection fraction (LVEF) compared with controls, and (**c**) DMD participants without late gadolinium enhancement (LGE) compared with controls

Figure 3 shows example native T1 maps and ECV maps, with corresponding LGE images. The mean native T1 value was significantly higher in DMD than in controls (1045 ms ± 57 vs 988 ms ± 14, *p* = 0.001) (Table 3, Fig. 4a). These differences remained significant when comparing DMD participants with normal LVEF (1039 ms ± 49 vs 988 ms ± 14, *p* = 0.002) and DMD participants without LGE (1038 ms ± 55 vs 988 ms ± 14, *p* = 0.011) (Fig. 4b, c).

**Table 3:** Difference in global native T1, ECV, and global T2 between Duchenne Muscular Dystrophy (DMD) participants and controls

	Control	DMD^a^	*p*-value*	DMD normal LVEF^b^	*p*-value*	DMD no LGE^c^	*p*-value*
	(*N* = 11)	(*N* = 31)		(*N* = 19)		(*N* = 12)	
Native T1 (ms)	988 ± 14	1045 ± 57	0.001	1039 ± 49	0.002	1038 ± 55	0.011
ECV^d^	0.24 ± 0.01	0.31 ± 0.05	<0.001	0.28 ± 0.03	<0.001	0.29 ± 0.03	0.001
	Control	DMD	*p*-value*				
	(*n* = 11)	(*n* = 12)					
T2 (ms)	49 ms ± 1	47 ± 2	<0.001				

*p-values for comparison with controls using Wilcoxon rank-sum

^a^Duchenne muscular dystrophy (DMD)

^b^Left ventricular ejection fraction (LVEF)

^c^Late gadolinium enhancement (LGE)

^d^Extracellular volume fraction (ECV)

**Fig. 5:**
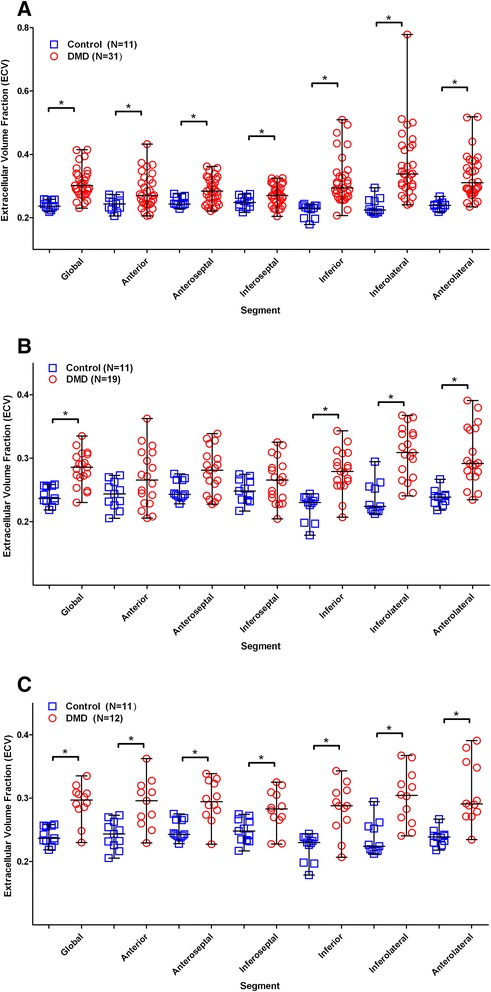
Scatterplots of global and segmental Extracellular Volume Fraction (ECV) values in Duchenne Muscular Dystrophy (DMD) and controls. **a** All DMD participants compared with controls, (**b**) DMD participants with normal left ventricular ejection fraction (LVEF) compared with controls, and (**c**) DMD participants without late gadolinium enhancement (LGE) compared with controls

ECV values demonstrated excellent intra- and inter-observer agreement (ICC = 0.963 for inter-observer, ICC = 0.957 for intra-observer, *p* < 0.001 for both). When compared with controls, DMD participants had significantly increased ECV (0.31 ± 0.05 vs 0.24 ± 0.01, *p* < 0.001) (Fig. 5a, Table 3). When comparing LVEF-normal DMD participants with controls, this difference remained significant (0.28 ± 0.03 vs 0.24 ± 0.01, *p* < 0.001) (Fig. 5b). LGE-negative DMD participants had a significantly higher ECV than controls (0.29 ± 0.03 vs 0.24 ± 0.01, *p* = 0.001) (Fig. 5c). DMD participants with systolic dysfunction had a greater mean ECV than DMD participants with normal function (0.35 ± 0.05 vs 0.28 ± 0.03, *p* < 0.001). There was no significant difference in ECV between DMD participants with and without LGE (0.32 ± 0.05 vs 0.29 ± 0.03, *p* = 0.306). However, 3 of those participants had no LGE in the mid-ventricular slice position used for T1 mapping. When DMD subjects were divided into those with visible LGE at the mid-ventricular slice and those without, those with visible LGE had significantly elevated ECV (0.33 ± 0.05 vs 0.29 ± 0.03, *p* = 0.03). The mean ECV of the targeted ROIs of LGE was significantly higher than the ECV of DMD participants without LGE (0.51 ± 0.08 vs 0.29 ± 0.03, *p* < 0.001). The range of ECV in these targeted ROIs was 0.39 to 0.63. DMD participants had a higher within subject standard deviation of ECV compared with controls (0.039 vs 0.024, *p* < 0.001). DMD participants without LGE also had a higher within subject standard deviation of ECV compared with controls (0.033 vs 0.024, *p* = 0.001).

There was no correlation between duration of angiotensin converting enzyme inhibitor, angiotensin receptor blocker, or beta-blocker therapy and either native T1 or ECV in DMD participants. There was a trend towards a negative correlation between duration of corticosteroids and ECV (rho = −0.351, *p* = 0.053), but this did not reach statistical significance. There was no difference in ECV between DMD participants with more than 6 months of steroid therapy and those with less than 6 months of therapy. There was a negative correlation between ECV and heart rates (r = −0.463, *p* = 0.009) suggesting that higher heart rates are associated with lower ECV. However, when adjusting for use of beta-blockers or LVEF, this association was no longer significant. There was no significant association between native T1 and heart rate within DMD (*p* = 0.345). There was a significant negative correlation between heart rate and T2 (r = −0.64, *p* = 0.025), though DMD participants with heart rates less than 100 bpm also had T2 values below the mean of controls (47 ms vs 49 ms, *p* < 0001). Global ECV had a significant negative correlation with LVEF (rho = −0.618, *p* < 0.001).

When analyzing the entire cohort, linear regression demonstrated no change in the association between global ECV and diagnosis when correcting for age, height, weight, or heart rate (Table S1, Additional file 1). There was also no change in the association between native T1 and diagnosis when correcting for age, height, or heart rate (Table S2, Additional file 1). There was a correlation between weight and native T1 using multivariable regression with no association between native T1 and diagnosis. The significance of this finding is unclear.

## Discussion

**Fig. 6:**
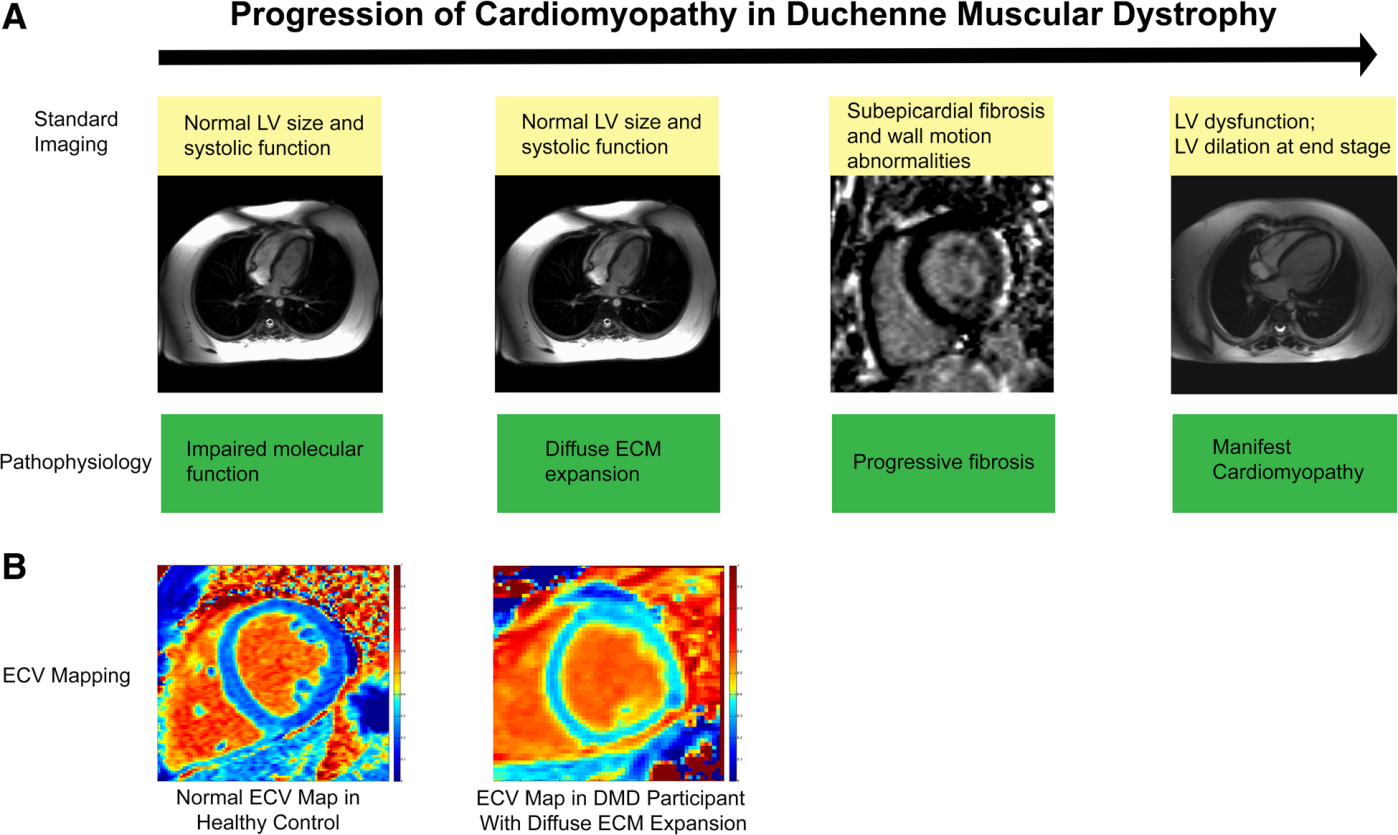
Duchenne muscular dystrophy (DMD) results in impaired molecular function prior to abnormal LV systolic function. Cardiomyopathy progresses with extracellular matrix (ECM) expansion, fibrosis, and manifest LV dysfunction. Standard imaging has limited ability to detect abnormalities before LV dysfunction (**a**). Extracellular volume fraction (ECV) mapping can provide a quantitative, early biomarker of DMD cardiomyopathy (**b**)

The abnormalities in native T1 and ECV are consistent with a diffuse expansion of ECM, a known pathological finding in DMD. These abnormalities were detectable even in participants without LGE. This has significance because native T1 and ECV mapping may provide early, non-invasive biomarkers of DMD myocardial disease (Fig. 6). This earlier detection may allow for the initiation of more timely disease specific therapy. ECV mapping also has potential to assess the efficacy of therapy before systolic dysfunction develops and may provide a surrogate outcome measure in clinical trials. This is the first manuscript of which we are aware that has comprehensively and prospectively evaluated native T1 and ECV mapping in DMD.

This study expands on our previous, retrospective work evaluating post-contrast T1 mapping [13]. The present prospective analysis suggests that native T1 and ECV are robust indicators of DMD myocardial disease. While native T1 values can be obtained without contrast administration, they are prone to site-to-site variability in the T1 mapping protocol, as well as inter-individual variability in heart rate. Because the ECV mapping protocol is ratiometric, the ECV measurement may be more generalizable across sites [10]. Which method better predicts morbidity and mortality will need to be explored. However, the results of this study strongly support the value of a systematic, prospective investigation of these potential biomarkers.

The increased within subject standard deviation of ECV suggests that there are areas of myocardium with less ECM expansion that are adjacent to areas of the myocardium with more ECM expansion. This increased variation is consistent with known ultrastructural findings in DMD [27]. This is the first time, to our knowledge, that a non-invasive imaging method has detected a potential corollary to these subtle ultrastructural changes. Whether this increased variability impacts morbidity, such as arrhythmia, remains to be determined in future studies.

The results of this study are similar to those of Florian et al., who evaluated patients with Becker muscular dystrophy (BMD) who were significantly older (mean age of 35 years vs 13.4 years in this study) [20]. The BMD patients without LGE did not have increased ECV compared with controls. In contrast, DMD subjects without cardiac involvement in our study still had elevated mean ECV. It is unclear if this represents a difference in the progression of Becker and DMD cardiomyopathy or if this is due to the larger number of patients without LGE in our study. The T1 and ECV values of DMD and control participants had significant overlap. This overlap was expected as the study enrolled DMD participants along the entire spectrum of disease (Fig. 6). Moreover, this overlap is desirable; identification of DMD participants with no ECM expansion will be equally important for research and clinical care.

The mean ECV value of all LGE-positive DMD participants was not significantly increased compared with that of LGE-negative DMD participants. This likely resulted from conservative ROIs and the variable distribution of LGE; not all DMD participants had LGE in the same slice location as the T1 mapping. When analysis was limited to DMD participants with LGE in the same slice location, the ECV in DMD participants with LGE was significantly increased compared to those without LGE. Moreover, the ECV in targeted ROIs of LGE was significantly elevated compared to participants without LGE.

Fatty infiltration and edema of DMD myocardium have the potential to affect T1 mapping and ECV values [27, 28]. None of the participants had chest pain or other cardiac complaints at the time of their CMR, so the likelihood that superimposed myocarditis is confounding our interpretation seems low. Myocardial fat and edema were not detected qualitatively using a double inversion recovery turbo fast spin echo nor quantitatively using T2 mapping. No patient in the study had visible fat or edema on these images. Of note, 5 DMD participants had native T1 values less than 900 ms in 6 total segments. These lower values did not appear to be due to contamination with epicardial fat and no myocardial fat was visible in T2-weighted imaging, though further evaluation for myocardial fat deposition may be warranted in future studies. While lower T2 values may have been affected by heart rate, DMD participants with heart rates less than 100 bpm had T2 values below the mean of controls. These findings, paired with the high ECV within areas of LGE, suggest that the larger areas of LGE in this study were due to fibrosis and not edema or inflammation.

Pathological studies in DMD suggest that diffuse microscopic fibrosis is present in most boys, and authors have suggested that this fibrosis precedes manifest systolic dysfunction [8, 29]. While we hypothesize that larger focal areas of fibrosis on LGE images are irreversible, studies suggest that this diffuse microscopic fibrosis can improve with therapy, creating a window of opportunity for earlier therapy in DMD [30–32]. ECV mapping allows detection of this diffuse fibrosis and ECV values correlate well with histological specimens [16, 17, 33, 34]. ECV has also been linked to outcomes in other disease processes [19, 35]. However, before its widespread adoption in clinical care or as an outcome measure, further evaluation in DMD must be performed. Serial follow up is necessary to assess the change in ECV over time. An association between ECV and DMD arrhythmia or mortality will need to be established and documentation of stabilization or improvement in ECV with medical therapy is essential. Our data suggest, however, that native T1 and ECV mapping in DMD are promising, non-invasive biomarkers of this progressive myocardial disease.

### Limitations

This is a single center study and in principle, this could make the results less generalizable. The Neuromuscular-Cardiology Clinic at our institution, however, draws DMD patients from across the country, and a large percentage of DMD patients are followed in a small number of centers similar to ours. The primary objective of this study was the detection of ECM expansion and not the identification of modifiers of this expansion; therefore, adjustment for medications was not performed. Moreover, because the study included control participants not taking any medications, the analysis could not be adjusted for current medications. We evaluated the effects of medications within the DMD population, though this analysis was likely underpowered, and this adjustment will be important for any analysis performed exclusively in DMD participants. Studies with a larger numbers of patients, likely multi-center, will be necessary to determine the effects of medications on ECM expansion, but this is beyond the scope of the current manuscript. The heart rates were significantly faster in DMD subjects. However, data suggest that the heart rate effects on T1 mapping are minimized with the sequence parameters used in this study [22]. Interestingly, there was a significant negative correlation between heart rate and ECV, suggesting that higher heart rates were associated with lower ECV. While this may have made it more difficult to achieve statistical significance, we suspect that this association is related to confounding from severity of disease; the association was no longer significant after correction for either beta-blocker usage or LVEF. There was no significant correlation between heart rate and native T1. The T2 values of DMD participants did correlate with heart rate and this may have led to some bias. The negative association between weight and native T1 may be related to myocardial fat deposition and should be explored in future studies.

Gadolinium contrast agents were not administered to healthy children in order to minimize the potential for adverse events. Therefore, the ages of the control group did not match perfectly with that of the DMD group. The progressive nature of DMD cardiomyopathy, however, suggests that older DMD participants are at higher risk of ECM expansion. Moreover, ECV does not vary significantly with age in healthy controls [36]. Because of the difficulty in obtaining CMR without anesthesia or sedation in young children, the youngest age of DMD participants was 8 years. The reported ECVs were only in the 6 myocardial segments at the mid portion of the LV; we anticipate that increasing the myocardial coverage will improve sensitivity.

## Conclusions

Our data demonstrate that native T1 and ECV maps can detect subclinical ECM expansion in DMD participants. These techniques may provide much needed surrogate outcome measures in DMD cardiovascular disease.

## Additional file

Additional file 1: Table S1. Linear regression of association between global Extracellular Volume Fraction and diagnosis (Duchenne Muscular Dystrophy = 1 vs Control = 2) controlling for age, height, weight, or heart rate. **Table S2.** Linear regression of association between global native T1 and diagnosis (Duchenne Muscular Dystrophy = 1 vs Control = 2) controlling for age, height, weight, or heart rate. (DOCX 47 kb)

## Abbreviations

bSSFP: Balanced steady state free precession
CMR: Cardiovascular magnetic resonance
DMD: Duchenne muscular dystrophy
ECG: Electrocardiogram
ECM: Extracellular matrix
ECV: Extracellular volume fraction
LGE: Late gadolinium enhancement
LV: Left ventricular
LVEF: Left ventricular ejection fraction
MOLLI: Modified Look-Locker inversion recovery
T1: Longitudinal relaxation time constant
