# Supplementary material for: Increased myocardial native T1 and extracellular volume in patients with Duchenne muscular dystrophy
Source: J Cardiovasc Magn Reson. 2016 Jan 21;18:5. doi: 10.1186/s12968-016-0224-7 (PMC4722665; doi:10.1186/s12968-016-0224-7)
Supplement: Additional file 1: Table S1. — Linear regression of association between global Extracellular Volume Fraction and diagnosis (Duchenne Muscular Dystrophy = 1 vs Control = 2) controlling for age, height, weight, or heart rate. Table S2. Linear regression of association between global native T1 and diagnosis (Duchenne Muscular Dystrophy = 1 vs Control = 2) controlling for age, height, weight, or heart rate. (DOCX 47 kb) [file 12968_2016_224_MOESM1_ESM.docx]

Supplemental Table 1. Linear Regression of Association Between Global Extracellular Volume Fraction and Diagnosis (Duchenne Muscular Dystrophy = 1 vs Control = 2) Controlling for Age, Height, Weight, or Heart Rate

|  | p-value |
| --- | --- |
| Diagnosis | <0.001 |
| Age | 0.243 |
| Diagnosis | 0.030 |
| Height | 0.356 |
| Diagnosis | 0.007 |
| Weight | 0.168 |
| Diagnosis | <0.001 |
| Heart Rate | 0.005 |

Supplemental Table 2. Linear Regression of Association Between Global Native T1 and Diagnosis (Duchenne Muscular Dystrophy = 1 vs Control = 2) Controlling for Age, Height, Weight, or Heart Rate

|  | p-value |
| --- | --- |
| Diagnosis | 0.031 |
| Age | 0.440 |
| Diagnosis | 0.023 |
| Height | 0.670 |
| Diagnosis | 0.320 |
| Weight | 0.010 |
| Diagnosis | 0.005 |
| Heart Rate | 0.303 |
